# Correlates of Objective Social Isolation from Family and Friends among Older Adults

**DOI:** 10.3390/healthcare6010024

**Published:** 2018-03-03

**Authors:** Linda M. Chatters, Harry Owen Taylor, Emily J. Nicklett, Robert Joseph Taylor

**Affiliations:** 1 School of Public Health and School of Social Work, University of Michigan, Ann Arbor, MI 48109, USA; chatters@umich.edu; 2 The Brown School of Social Work, Washington University in St. Louis, St. Louis, MO 63130, USA; hotaylor@wustl.edu; 3 School of Social Work, University of Michigan, Ann Arbor, MI 48109, USA; enicklet@umich.edu; 4 School of Social Work and Institute for Social Research, University of Michigan, Ann Arbor, MI 48109, USA

**Keywords:** African-American, Afro-Caribbean, social support, extended family, kinship, support network

## Abstract

This study examined the correlates of objective social isolation from extended family members and friends among older adults. The analysis is based on the older adult sub-sample of the National Survey of American Life (*n* = 1321). Multinomial logistic regression analyses examined race/ethnicity, demographics, functional health and family and friend network factors as correlates of objective isolation from family and friends. Only 4.47% of respondents were objectively isolated from both their extended family and friends, 10.82% were isolated from their friends, and 7.43% were isolated from their family members. Men were more likely to be objectively isolated from both family and friends and older adults who live with others were significantly more likely to be objectively isolated from their friends. When controlling for subjective social isolation, the two measures of functional health were significantly associated with objective social isolation. In particular, higher levels of self-care impairment decreased the risk of being objectively isolated from friends only, whereas higher mobility impairment was associated with an increased likelihood of being objectively isolated from friends only. Subjective evaluations of social isolation from family and friends were consistently associated with being objectively isolated from family and friends. There were no significant differences between African-Americans, Black Caribbeans and non-Hispanic Whites in objective isolation. These and other findings are discussed in detail.

## 1. Introduction

Social isolation is a growing social problem in the United States [1,2,3] that has been recognized by several health professions, including Public Health [1] and Social Work [2]. One of the reasons why social isolation is such a critical issue is due to the numerous negative impacts that it has on the health and well-being of older adults [1,2]. Being socially isolated is associated with higher rates of all-cause mortality [3,4,5,6,7,8], chronic diseases [9], cognitive decline [10] and dementia [11]. For instance, the classic work of Berkman and Syme (1979) [8] found that the risk of mortality among adults with the fewest social ties was more than double the risk for those with the most social ties. Additionally, numerous articles have found that both objective and subjective social isolation are linked to a broad variety of poor health conditions, including cardiovascular disease, recurrent myocardial infarction, atherosclerosis, high blood pressure, cancer and delayed cancer recovery (see review by Umberson & Montez, 2010) [12]. The goal of this study is to investigate the correlates of objective social isolation from family and friends among a national sample of African-American, Black Caribbean, and non-Hispanic White older adults. The literature review begins with a discussion of the research on the impact of social isolation on health, followed by a review of research on family and friendship social support networks and race and ethnicity, in relation to social networks, social relationships, and social isolation. The literature review ends with a statement of the purpose of the present study.

### 1.1. Linkages between Social Isolation and Health

As noted previously, social isolation is negatively associated with a range of indicators of physical health (see reviews by Lubben et al., 2015 and Klinenberg, 2016) [1,2]. One likely pathway linking social isolation and poorer health status involves the extent to which being isolated limits access to informal social support systems that provide assistance and resources to promote health. Studies in gerontology, family studies, and health services research indicate that adult children, spouses, other family members, and friends are important for providing health relevant information and informal support to social network members. These informal support systems provide general health maintenance activities, direct in-home care (e.g., meal preparation, personal care), chronic disease and medication management, rehabilitative efforts, health decision-making, and assistance in accessing and navigating formal health resources (e.g., transportation, medical appointment scheduling) [13,14]. 

Informal health care of this sort is considerable in its scope, involving more than 43 million caregivers and had an estimated value of 470 billion dollars in 2013 [14]. Post-discharge health care planning increasingly relies on the existence and use of informal sources of health supports to provide home-based high intensity care for patients with complex medical needs [15]. Further, changes in the reimbursement structures for paid assistance frequently translate into greater reliance on unpaid informal care [16]. Unpaid informal care reduces medical care expenditure and delays nursing home placement [17] and, despite the high opportunity costs associated with informal care (lost wages), informal care remains a more economical alternative than formal skilled care services [18]. Finally, studies indicate that informal caregiving is predominately carried out by family members and close friends [13,14,15]. 

### 1.2. Family and Friend Social Networks

Our focus on objective isolation from family and friends acknowledges the fact that, as the most important primary groups in any society, family and friends are expected to fulfill the majority of our affiliative and social needs. Given these normative expectations, we anticipate that only a few individuals are likely to indicate that they are isolated from both groups. However, these individuals will be the most vulnerable with regard to health and mental health. Accounting for both family and friend relationships is important to address questions about whether and in what ways connections with these networks are differentially important for social isolation among older adults. Research on friendship relationships [19] suggests that friends are distinct from family in several respects. Friendships are voluntarily chosen, based on mutual interests and life experiences, and are likely to involve age peers. In contrast, family relationships are obligatory, based on notions of family responsibility and duty, and as a result, may entail issues of emotional ambivalence. Finally, given specific affinities and personal similarities found in friend relationships, friends provide different forms of support and interactions (e.g., confidant) than do family members [20,21,22]. 

### 1.3. Race/Ethnicity and Social Isolation

Social isolation is of particular relevance for elderly from racial and ethnic minorities for several reasons. First, racial and ethnic minority elderly, particularly African-American elders, are disproportionally affected by social, economic and health characteristics (e.g., lower levels of income, poorer health profiles, degraded neighborhood and community environments and reduced access to formal resources) that are recognized risk factors for social isolation [2,3,23]. Second, for African-American elderly and other racial and ethnic minority elderly who are typically underserved in terms of the formal health and social service sectors, family and friend informal social networks provide needed assistance and resources that offset social, economic and health deficits and are associated with psychological well-being [20,24,25,26]. Given these life circumstances, social isolation from family and friends may be particularly damaging for the health and emotional well-being of elderly from racial and ethnic minorities. 

Across the adult age range, African-Americans are less likely than non-Hispanic Whites to interact with friendship networks and to identify friends as an important source of support [19,27,28]. Studies of social network composition for older adults similarly indicate that African-American social networks are primarily populated by family members [29]. Research on African-American populations indicates that, in some instances, friendships are elevated to the status of fictive kin relationships [19,30]. Fictive kin are individuals who are unrelated by birth, marriage or adoption, but who are accorded many of the rights and responsibilities of family members. Recent research involving race and ethnic comparisons of fictive kin relations indicate that although African-Americans and Black Caribbeans are more likely to report having fictive kin relations and to have more fictive kin in their networks, non-Hispanic Whites report receiving social support more often from fictive kin [19]. 

An analysis comparing the family networks of Black Caribbeans and African-Americans found that, overall, they are relatively similar [19]. African-Americans (compared to Black Caribbeans) reported that they gave help to family members more often and were more likely to have daily interaction with family members. However, there were no significant differences between African-Americans and Black Caribbeans in the frequency of receiving support from family, the overall frequency of family interaction, the frequency of emotional support, the degree of subjective family closeness, and the number of family members who would provide assistance if needed [19]. 

Currently, there is extremely little research that has investigated racial and ethnic differences in social isolation. Although there are several articles that have investigated the quality and quantity of social support networks, there is little research that has investigated race differences in the lack of social ties. For instance, while several studies have found that African-Americans interact with their families more frequently than Whites (e.g., Taylor et al., 2013) [19], there is no work that we are aware of that has focused on race differences in the absence of social relationships. In addition, research regarding race differences in social support has been mixed, with some studies finding that African-Americans receive more support from family, others reporting no racial differences and others reporting that non-Hispanic Whites receive more support from family (see Taylor et al., 2013) [19]. Similarly, Taylor et al. (2013) [19] found that African-Americans are less involved in friendship networks than Whites, but Snowden (2001) [31] found the opposite. These contradictory findings are due to a variety of factors, including differences in (a) the age of sample populations studied (e.g., young mothers, adults, elderly adults), (b) the life circumstances of study populations (e.g., diabetes patients, single mothers), (c) whether support was examined in relation to a crisis versus everyday situations (e.g., emergencies, serious health problems, caregiving), and (d) the specific kin groups examined (e.g., parents, adult children, grandparent, non-kin) (see Sarkisian & Gerstel, 2004;Taylor et al., 2013) [19,32]. 

### 1.4. Purpose of the Study

The present study investigates a broad set of sociodemographic, health, and family and friend network factors as correlates of objective social isolation from family and friends within a national sample of older African-Americans, Black Caribbeans and non-Hispanic Whites. The study makes several important contributions. First, whereas prior work on social isolation primarily focuses on family networks and interactions, this study incorporates information on social isolation from both family and friends. Second, we use multivariate analyses to determine the independent effects of sociodemographic and health factors that are identified as risk factors and correlates of objective isolation. Third, we examine the independent impacts of qualitative features of family and friend networks as correlates of different patterns of objective isolation. Finally, our analyses are conducted using a sample of older adults from three racial/ethnic groups to assess potential overall differences in patterns of objective social isolation. In addition, the ability to simultaneously control for relevant factors contributes to the broader literature by providing a clearer understanding of the independent effects of relevant correlates on objective isolation.

Drawing on previous research, we anticipate that demographic correlates associated with being objectively socially isolated from family and friends (lack of contact and interaction) will include being of older age, male gender, being unmarried, having lower levels of education and income, and living alone. Given conflicting evidence regarding the relationship between race and ethnicity and social isolation, specific hypotheses for this factor are not proposed. We anticipate that elders who rate their health as poor or experience impairments in functional status (i.e., mobility and self-care) will report being objectively socially isolated. Finally, older persons who report being subjectively socially isolated (i.e., not feeling emotional close to family and friends) will report being objectively socially isolated. This is consistent with previous research which has indicated that levels of affection with members of social networks are associated with the levels of interaction with them [21,24]. 

## 2. Methods

### 2.1. Sample

The National Survey of American Life: Coping with Stress in the 21st Century (NSAL) was conducted by the Program for Research on Black Americans at the University of Michigan’s Institute for Social Research [33]. The field work for the study was completed by the Institute for Social Research’s Survey Research Center, in cooperation with the Program for Research on Black Americans. A total of 6082 interviews were conducted with persons aged 18 or older, including 3570 African-Americans, 891 non-Hispanic Whites, and 1621 Blacks of Caribbean descent. Among persons 55 years of age and older, 837 were African-American, 298 were non-Hispanic Whites and 304 were Caribbean Blacks, to give a total of 1439 people aged over 55 years of age. This older sub-sample was used in this study. After accounting for missing data, the analytic sample for this study was 1321. 

The NSAL sample was based on a national multi-stage probability design with an overall response rate of 72.3%. Respondents were compensated for their time. The data collection was conducted from 2001 to 2003. Final response rates for the NSAL two-phase sample designs were computed using the American Association of Public Opinion Research (AAPOR) guidelines (for Response Rate 3) [34] (see Jackson et al., 2004 [33] for a more detailed discussion of the NSAL sample). The NSAL data collection was approved by the University of Michigan Institutional Review Board (IRB #B03-00004038-R1). The NSAL is publicly available through the Inter-University Consortium of Political and Social Research at the University of Michigan. 

### 2.2. Measures

#### 2.2.1. Dependent Variable

The dependent variable is a measure of objective social isolation from family and friends that was created by combining the frequency of contact with family and the frequency of contact with friends. The frequency of contact with family members was measured by the question, “How often do you see, write or talk on the telephone with family or relatives who do not live with you? Would you say nearly every day, at least once a week, a few times a month, at least once a month, a few times a year, hardly ever or never?” This same question was also asked of friends (i.e., friend contact). Both questions were recoded by combining the response categories: (1) nearly every day, at least once a week, a few times a month vs. (2) at least once a month, a few times a year, hardly ever or never. This resulted in two binary variables: objectively isolated from family (Yes/No) and objectively isolated from friends (Yes/No). These variables were then combined into a single four-category pattern variable reflecting the respondents who were (1) objectively isolated from both family and friends, (2) objectively isolated from family only, (3) objectively isolated from friends only, or (4) not objectively isolated from family and friends. This approach provides a more nuanced assessment of objective social isolation and permitted us to examine source-specific patterns of objective social isolation [21]. 

#### 2.2.2. Independent Variables

Sociodemographic factors (i.e., age, race/ethnicity, gender, family income, education, marital status, household status) were utilized as independent variables. Age was coded as a continuous variable. Race/ethnicity was coded as African-American, Black Caribbean and non-Hispanic White. Missing data for household income were imputed for 773 cases (which is 12.7% of the total NSAL sample) and missing data for education were imputed for 74 cases of the total NSAL sample. Imputations were completed using an iterative regression-based multiple imputation approach, incorporating information about age, sex, region, race, employment status, marital status, home ownership, and nativity of household residents. Age and education were coded in years. Marital status was coded as married/cohabiting and not married. Household size was coded as living alone vs. living with at least one other person.

Two variables for functional status (disability) were included in this analysis using the World Health Organization Disability Assessment Schedule (WHO-DASII) [35] in which participants were asked about their functional ability in the past 30 days with respect to self-care (e.g., washing, dressing. caring for oneself) and mobility (e.g., mobility within the home, standing for 30 min, and walking a distance). Items assessed the number of days of impairment in each domain, weighted by self-assessed difficulty in performing activities. Scores in each of the two domains were transformed to range from zero (no impairment) to one hundred (complete impairment). The analysis included a measure of self-rated physical health: How would you rate your overall physical health at the present time? Would you say it is excellent, very good, good, fair or poor? Response categories ranged from poor (1), fair (2), good (3) and very good (4), to excellent (5). In the multivariate analysis, mobility and self-care were divided by 10 and income was divided by 5000 in order to provide a better understanding of their net impact. 

Finally, we were also interested in whether qualitative aspects of family and friend relationships would emerge as correlates of objective social isolation. The NSAL dataset contains measures of subjective isolation from family and friends. Subjective isolation from family was measured by the question: “How close do you feel towards your family members? Would you say very close, fairly close, not too close or not close at all?” This item was also asked of friends (i.e., subjective isolation from friends). Values for response categories for both variables were not close at all (4), not too close (3), fairly close (2) and very close (1).

### 2.3. Analysis Strategy

One-hundred and eighteen respondents with missing data on any variable were excluded from analyses. Self-rated physical health (70 cases) and the two functional status variables (69 cases) had the most missing data. The use of list-wise deletion in cases where missing data represents less than 10% of the sample is considered to be acceptable, having little impact on the validity of statistical inferences [36]. Multinomial logistic regression was used to analyze the data. The reference category was “not being objectively isolated from family and friends”. Specifically, the results focus on contrasts involving four distinct groups: (1) objectively isolated from both family and friends vs. not objectively isolated, (2) objectively isolated from friends vs. not objectively isolated, (3) objectively isolated from family vs. not objectively isolated.

For the multinomial logistic regression analyses, relative risk ratios (RRR) and 95% confidence intervals are presented. The analyses were conducted using Stata which uses the Taylor expansion approximation technique for calculating the complex design-based estimates of variance. To obtain results that are generalizable to the population, all analyses utilized analytic weights. All statistical analyses accounted for the complex multistage clustered design of the NSAL sample, and unequal probabilities of selection, nonresponse, and poststratification, to calculate weighted, national representative population estimates and standard errors. All percentages reported are weighted.

## 3. Results

Descriptive data for all study variables are presented in Table 1. Overall, 77.28% of older adults in our sample indicated that they are not objectively isolated from their extended family or friends. Only 4.47% are objectively isolated from both their extended family and friends, 10.82% are objectively isolated from their friends, and 7.43% are objectively isolated from their family members. Respondents reported low levels of subjective isolation from family (mean = 1.3) and friends (mean = 1.61). The profile of sociodemographic and functional status factors indicates that 39% of this sample of older adults are African-Americans, 58% are non-Hispanic Whites and 2.6% are Black Caribbeans. Any given individual in the sample is more likely to be female, not married or cohabiting, and the average age was 66.5 years. Respondents reported an average of 12 years of education, have family incomes of roughly $37,000, and 41% reported that they live alone. With regard to functional health status, respondents’ average level of mobility impairment is 7.98 and average self-care limitation is 1.44, indicating a low level of functional limitations in this sample. The average for self-rated health was 3.12 indicating the sample assessed their overall health as being ‘good.’

Table 2 presents the multinomial logistic regressions of the demographic, health status, and subjective isolation variables on objective isolation from extended family and friends. In all regressions, the comparison category is “not objectively isolated from family and friends”. Two sets of regression models are presented. The first set (Models 1, 2, and 3) includes the demographic factors and functional status as independent variables. The second set (Models 1a, 2a, and 3a) includes the demographic factors, health status variables, and the two subjective isolation variables. Models 1 and 1a contrast “being objectively isolated from both family and friends” against the comparison category “not objectively isolated from family and friends”. An examination of the relative risk ratios for Model 1 revealed that gender is significantly associated with being objectively isolated from both family and friends. Men (RRR = 5.56, *p* < 0.001) are more likely to be objectively isolated from both groups. With the inclusion of subjective isolation from family members and subjective isolation from friends in Model 1a, gender remains significant. The two subjective isolation variables are also significant, indicating that older adults who are subjectively isolated from their family members (RRR = 3.40, *p* < 0.001) and those who are subjectively isolated from their friends (RRR = 4.56, *p* < 0.001) are significantly more likely to be objectively isolated from both family and friends. 

Models 2 and 2a contrast respondents who are not objectively isolated from family and friends with those who are objectively isolated from their friends only. In Model 2, household size (RRR = 2.45, *p* < 0.05) and age (RRR = 0.97, *p* < 0.05) are associated with objective isolation from friends, and these relationships remain significant in Model 2a. Older adults who live with others are significantly more likely to be objectively isolated from their friends, and age is negatively associated with being objectively isolated from friends. 

In Model 2a, the two functional health status variables now achieve significance. Increasing self-care impairment (RRR = 0.77, *p* < 0.05) decreases the risk of being objectively isolated from friends only (compared to not being objectively isolated from both family and friends) and higher mobility impairment (RRR = 1.21, *p* < 0.01) is associated with an increased likelihood of being objectively isolated from friends only. Higher subjective isolation from friends (RRR = 3.80, *p* < 0.001) is positively associated with an increased likelihood of being objectively isolated from friends.

The analyses comparing respondents who are not objectively socially isolated from family and friends with those who are objectively isolated from their family members only are presented in Models 3 and 3a. Three significant relationships for gender, family income, and living arrangements are noted and retain significance in Model 3a. In particular, men (RRR = 2.69, *p* < 0.001) and respondents who live with others (RRR = 2.03, *p* < 0.05) are more likely to be objectively isolated from family only (compared to not objectively isolated from family and friends). On the other hand, those with higher family incomes (RRR = 0.89, *p* < 0.05) are less likely to be objectively isolated from family members only. Older respondents (RRR = 1.05, *p* < 0.05) are more likely to be objectively isolated from family only. Persons who are subjectively isolated from their family members (RRR = 7.79, *p* < 0.001) are significantly more likely to be objectively isolated from their family members, whereas those who are subjectively isolated from their friends (RRR = 0.49, *p* < 0.01) have a lower likelihood of being objectively isolated from family only. 

Finally, there were no significant race/ethnicity, educational or marital status differences in any of the regression models.

## 4. Discussion

Social isolation is an important issue affecting older adults, with implications for their levels of social support, as well as their physical and mental health. As noted earlier, older adults who are socially isolated have poorer mental health, physical health and higher rates of mortality [3,5,6,7,8,9,31] underscoring the importance of this issue. This study provided a comprehensive assessment of objective social isolation from family and friends among older adults. Study advantages included the ability to examine these relationships within a large national sample of racially and ethnically diverse older adults and the use of multivariate analyses to statistically control for demographic and other factors that are known to be associated with social networks and relationships. Overall, our analysis found that only 4.47% older adults in our sample were objectively isolated, whereas three out of four (77.28%) older adults were not objectively isolated from both their extended family and friends. Our finding that 22.72% of respondents report being objectively isolated from family, friends or both is consistent with previous prevalence estimates for social isolation (unspecified as objective or subjective) among older adults, which range between 15% and 40% [23]. 

Consistent with research on social support and social networks [29,37,38], we found that women were less likely than men to be objectively isolated from both family and friends and less likely to be objectively isolated from family only. These findings are consistent with a broad literature, which indicates that women are more invested in, and connected to, social networks, including family and friends [37]. Women’s lifelong investments in family and friend interactions and relationships [39], including their social roles as caregivers to others [40], may mean that they are less likely to experience social isolation in older age. As these and other data [29,38,41] demonstrate, older men have fewer social resources and limited social interactions with others, making them vulnerable with respect to objective social isolation. These differences highlight the importance for understanding objective social isolation in relation to the particular roles of gender in social network experiences that characterize the lives of older women and men [37]. 

With respect to age, in this sample of adults who are 55 years of age and above, being older was associated with being less likely to be objectively isolated from friends only. On the other hand, older age was associated with being more likely to be objectively isolated from family only. Both effects only emerged within the context of the enhanced regression models that controlled for subjective social isolation from family and friends, indicating that the effects of age are only revealed after accounting for the independent effects of family and friend subjective isolation. These findings indicate that, in contrast to the conventional view of older age as a risk factor for social isolation, the reality is more complex than previously thought and depends on the specific social group (family vs. friends) in question. Although with cross-sectional data we can only speculate, this differential pattern may indicate that growing older is associated with transitions in social contact and interaction with family versus friends, with persons of very advanced age having increasing contact with friends as opposed to family. For persons of very advanced age, this transition could be partially due to losses of older family members due to death (aunts, uncles, cousins and siblings), as well as opportunities to create new friendships. The current findings also indicate that older adults with higher family incomes were less likely to be objectively isolated from family only, suggesting that higher financial resources (possibly reflecting one’s ability to afford social and leisure activities with family) are important in protecting against objective isolation from family.

The sociodemographic factors of race/ethnicity, marital status, and education were not associated with objective isolation from family and friends. Prior research findings on race and ethnic differences in the composition and size of social support networks are equivocal due to a variety of conceptual and methodological issues (e.g., measures used, study populations) found in disparate studies [19,32]. Further, prior work suggests that marriage and higher education have specific social benefits in terms of larger and more integrated social networks [42,43]. However, the present study of older African-Americans, Black Caribbeans and non-Hispanic Whites employed identical dependent and independent measures within a multivariate context to assess the independent relationships between correlates and social isolation. Accordingly, the absence of significant effects for these factors indicates that the older adults in this sample, who differed on race/ethnicity, marital status, and education, were comparable with regard to objective isolation from family and friends.

Living arrangements (alone or with others) are often interpreted as a straightforward indicator of social isolation (see review by Cacioppo & Cacioppo, 2014 [3]). That is, living with others is presumed evidence that one is socially engaged, while living alone presumes a state of being socially isolated. Our findings, however, demonstrated that living with others was associated with different patterns of objective isolation from family and friends. Specifically, older adults who lived with others were more likely to report being objectively isolated from friends only and family only [28]. Stated another way, respondents who lived by themselves (contrasted to those who lived with others) had a lower likelihood of being objectively isolated from friends only and from family only. In essence, our findings indicated that living arrangements themselves—alone or with others—were not indicative of social contact or engagement. 

Impairments in the areas of self-care and mobility were both associated with objective isolation from friends only; however, their impacts differed. Greater mobility impairment (i.e., moving about in one’s home, standing for 30 min, walking a long distance) was associated with a greater likelihood of being objectively isolated from friends. In contrast, greater impairment in self-care (i.e., bathing, dressing, caring for oneself) was associated with a lower likelihood of isolation from friends. The findings suggest that it is important to distinguish between types of impairments and functionality and their association with objective isolation from friends. Mobility impairments, in particular, may represent a significant risk factor for objective isolation from friends due to their potential for limiting one’s ability to pursue social activities outside the home. Impairments in self-care, which were associated with a reduced likelihood of objective isolation from friends, may operate differently with respect to social engagement with friends. Self-care impairments are potentially important in indicating the need for social care and assistance from others and in activating support from and interaction with one’s social network. Older adults with greater impairment in self-care may have befriended their paid caregivers, which would explain a greater frequency of contact and reduced objective isolation from friends. Another distinct possibility is individuals with greater self-care impairment could be closer to mortality, and therefore have more friends visiting them as they become more ill. This would increase their engagement and decrease their objective isolation from friends. It is important to note that these two functional impairment variables were only significant when controlling for the two subjective isolation variables. This indicates the possible presence of a suppression effect.

As one might expect, both subjective isolation from family and subjective isolation from friends had particularly strong relationships with our measures of objective isolation. Subjective isolation from family was a risk factor for being objectively isolated from both family and friends and from family only. Similarly, subjective isolation from friends was a risk factor for being objectively isolated from both family and friends, as well as from friends only. These findings indicate that subjective isolation may be a precursor to objective isolation. That is, when individuals do not feel affectively close to their family and friends they are significantly less likely to interact with them. 

An interesting finding in this study was that feeling subjectively isolated from friends was associated with a lower likelihood of being objectively isolated from family only. In essence, older adults who reported lower levels of closeness in their friend relationships interacted more frequently with family members. This finding is similar to research on support network typologies [44,45] that uses person-centered analysis techniques (e.g., latent class analysis) to derive distinct social network typologies. The resulting typologies reflect the relevant network group that is represented (e.g., family, friends, church members), as well as the associated characteristics of the network (e.g., affective dimensions, contact, negative interaction). Findings from these studies of adults in the US and other countries [44] have identified the family-focused social network type which is characterized by high integration within family networks coupled with significantly lower integration with non-family networks. This family-focused social network type is consistent with the findings in this paper. In relation to research on social isolation, these collective findings underscore the importance of understanding and assessing both objective features of social relationships (e.g., level of contact), and subjective evaluations of the quality of social relationships (i.e., perceived isolation), as well as investigating family and friend social networks.

### 4.1. Implications for Research and Practice

The study’s findings have several implications for research and practice concerning social isolation of older adults. First, living alone is often identified in research as a risk factor for both objective and subjective social isolation. However, our findings demonstrate that solitary living, per se, is not evidence of being socially isolated in its absolute sense. Living alone should not be conflated with the absence of all social contact and, in fact, a range of social connections and relationships may be associated with different types of living arrangements. Following from this, it is important to recognize that living arrangements, whether alone or with others, are the result of various decisions, constraints, transitions, and circumstances that may not be voluntary or under individual control. For example, in some instances, solitary living is the result of an intentional and planned choice to live by oneself while maintaining social interactions with family and friends who reside elsewhere. 

Conversely, although sharing a household means direct contact with others, this living arrangement may be the result of constrained options and/or lack of choice. Under these circumstances, transitioning to a shared housing arrangement may bring about changes in social contact and interactions with others. For example, an older person who moves in with others may have to relocate to a different community or city, thereby losing direct contact with neighbors, friends and other peers (e.g., church members). An older person who is a household head with custodial responsibilities for a grandchild(ren), may experience reduced levels of contact with family and friends as a result of caregiving duties and responsibilities. Given the complex circumstances surrounding different types of living arrangements, social isolation assessments and direct practice interventions with older adults must be particularly sensitive to how and in what specific ways household status is associated with social engagement with family and friends [46]. 

### 4.2. Study Limitations

Several study limitations must also be considered. First, on average, most people in the sample were not objectively socially isolated. As a consequence, our analysis contrasting different patterns of objective isolation from family and friends involved relatively small numbers of cases for the isolated categories. The NSAL is the only national probability survey of African-Americans and Black Caribbeans; however, the timeliness of the data, collected between 2001 and 2003, is a limitation. Finally, as noted in the discussion, several of the observed relationships involved changes in specific patterns of objective isolation from family and friends (e.g., age differences) and the circumstances surrounding reported living arrangements (i.e., alone vs. with others). These and other questions are suggestive of a causal sequence of events that cannot be addressed with cross-sectional data and require more in depth, prospective information concerning the processes related to social isolation.

Despite these limitations, the significant advantages of the sample and the diverse study variables provided a unique opportunity to examine sociodemographic, health status and qualitative features of family and friend relationships and their associations with objective social isolation from family and friends among older adults. As an initial effort to better understand how different factors are associated with social isolation within diverse groups of older adults, this study revealed different patterns of objective isolation in relation to gender, age, living arrangements, functional health status, and subjective isolation from family and friends. The findings of group differences in objective social isolation argue for further exploration of patterns of objective isolation among older adults that focus on within-group differences (e.g., gender). Given the importance of social isolation as a significant public health and social welfare issue for older adults, it is important to clearly define and understand the scope and patterns of social isolation in order to plan effective strategies, interventions and policies to address the problem [1,2].

## 5. Conclusions

In recent years, social isolation has emerged as a significant public health and social welfare issue impacting older adults. The development of strategies, interventions, and social policies combatting social isolation must be built upon solid research that identifies the distribution and patterns of social isolation within racially and ethnically diverse groups of elders. Priority tasks include identifying risk and protective factors for social isolation (i.e., objective and subjective) that are associated with specific demographic and health characteristics of older adults, the quality and frequency of social contacts with others, and the characteristics of elders’ living arrangements. Exploring patterns of social isolation in relation to both family and friend relationships is important for assessing a wider group of available social contacts. Further, focusing on both family and friend connections provides a better understanding of the complex circumstances (i.e., decisions, constraints, preferences) that shape patterns of social isolation and social engagement and their relationships with older adults’ well-being.

## Figures and Tables

**Table 1:** Demographic Characteristics of the Sample and Distribution of Study Variables.

	%	n	Mean	SD	Min	Max
Objective Social Isolation						
Socially Isolated from Both	4.47	54				
Socially Isolated from Family	7.43	86				
Socially Isolated from Friends	10.82	133				
Not Socially Isolated	77.28	1048				
Subjective Isolation From Family		1321	1.30	0.61	1	4
Subjective Isolation From Friends		1321	1.61	0.76	1	4
Race/Ethnicity						
African-American	39.32	756				
Black Caribbean	2.63	281				
Non-Hispanic Whites	58.05	284				
Gender						
Male	44.25	496				
Female	55.75	825				
Age		1321	66.54	8.91	55	93
Family Income		1321	37,416	40,195	0	640,000
Education		1321	12.12	3.44	0	17
Marital Status						
Married/Cohabit	46.85	474				
Non-Married	53.15	847				
Household Size						
Live Alone	41.50	682				
Live with Someone Else	58.50	639				
Mobility		1321	7.98	19.54	0	100
Self Care		1321	1.44	8.89	0	100
Self Rated Health		1321	3.12	1.15	1	5

Notes: Percentages and n are presented for categorical variables and means and standard deviations are presented for continuous variables. Percentages are weighted and frequencies are un-weighted.

**Table 2:** Weighted Multinomial Logistic Regressions of Objective Social Isolation Among Older Adults (*n* = 1321) in the National Survey of American Life (NSAL; 2001–2003).

	Socially Isolated from Both Family and Friends ^1^		Socially Isolated from Friends Only ^1^		Socially Isolated from Family Only ^1^	
	Model 1	Model 1a	Model 2	Model 2a	Model 3	Model 3a
	RRR (95% CI)	RRR (95% CI)	RRR (95% CI)	RRR (95% CI)	RRR (95% CI)	RRR (95% CI)
Race/Ethnicity ^2^						
African-American	1.00	1.00	1.00	1.00	1.00	1.00
Black Caribbean	1.21 (0.41, 3.61)	1.78 (0.48, 6.66)	1.98 (0.83, 4.68)	2.09 (0.94, 4.64)	1.57 (0.48, 5.15)	1.92 (0.56, 6.54)
White	1.33 (0.49, 3.58)	1.27 (0.49, 3.31)	1.37 (0.79, 2.37)	1.31 (0.73, 2.36)	1.63 (0.99, 2.67)	1.42 (0.75, 2.69)
Gender						
Male	5.56 (2.49, 12.40) ***	3.47 (1.55, 7.77) **	1.82 (0.86, 3.85)	1.56 (0.72, 3.40)	2.69 (1.44, 5.02) **	2.75 (1.71, 4.44) ***
Female	1.00	1.00	1.00	1.00	1.00	1.00
Age	1.01 (0.94, 1.10)	1.03 (0.97, 1.10)	0.97 (0.94, 1.00) *	0.97 (0.94, 1.00) *	1.01 (0.96, 1.06)	1.05 (1.00, 1.10) *
Family Income	1.00 (0.96, 1.05)	0.99 (0.94, 1.04)	0.92 (0.85, 1.00)	0.92 (0.85, 1.01)	0.89 (0.83, 0.96) **	0.93 (0.87, 0.99) *
Education	1.06 (0.95, 1.19)	1.00 (0.83, 1.20)	0.99 (0.91, 1.06)	0.96 (0.89, 1.03)	1.07 (0.95, 1.21)	1.04 (0.93, 1.15)
Marital status						
Married	0.57 (0.16, 1.95)	0.87 (0.20, 3.81)	0.85 (0.41, 1.78)	0.90 (0.42, 1.95)	0.86 (0.41, 1.79)	1.02 (0.49, 2.14)
Non-Married	1.00	1.00	1.00	1.00	1.00	1.00
Household Size						
Live Alone	1.00	1.00	1.00	1.00	1.00	1.00
Live with Others	2.24 (0.50, 9.96)	2.00 (0.43, 9.25)	2.45 (1.08, 5.53) *	2.54 (1.06, 6.11) *	2.03 (1.08, 3.79) *	2.16 (1.01, 4.63) *
Mobility	1.07 (0.88, 1.31)	1.14 (0.87, 1.49)	1.12 (0.97, 1.30)	1.21 (1.07, 1.36) **	1.02 (0.86, 1.20)	1.00 (0.82, 1.22)
Self-Care	0.87(0.53, 1.42)	0.83 (0.47, 1.45)	0.84 (0.67, 1.05)	0.77 (0.60, 1.00) *	0.99 (0.76, 1.29)	1.05 (0.80, 1.38)
Self Rated Health	1.08 (0.65, 1.78)	1.34 (0.79, 2.26)	1.10 (0.79, 1.54)	1.27 (0.96, 1.68)	0.98 (0.59, 1.64)	0.96 (0.59, 1.55)
Subjective Isolation From Family	--	3.40 (1.99, 5.82) ***	--	0.83 (0.43, 1.60)	--	7.79 (5.51, 11.01) ***
Subjective Isolation From Friends	--	4.56 (2.50, 8.30) ***	--	3.80 (2.75, 5.26) ***	--	0.49 (0.29, 0.84) **

Note: RRR = relative risk ratio, CI = confidence intervals; ^1^ not socially isolated is the comparison group, ^2^ Several independent variables are represented by dummy variables; for race/ethnicity African-American is the excluded category; for gender, 0 = female, 1 = male; for marital Status, non-married is the excluded category; for household size, living alone is the excluded category; * *p* < 0.05, ** *p* < 0.01, *** *p* < 0.001.
